# Probing substrate influence on graphene by analyzing Raman lineshapes

**DOI:** 10.1186/1556-276X-9-64

**Published:** 2014-02-07

**Authors:** Chen-Han Huang, Hsing-Ying Lin, Cheng-Wen Huang, Yi-Min Liu, Fu-Yu Shih, Wei-Hua Wang, Hsiang-Chen Chui

**Affiliations:** 1 Center for Nano Bio-Detection, National Chung Cheng University, Chiayi 621, Taiwan; 2 Department of Photonics, National Cheng Kung University, Tainan 70101, Taiwan; 3 Institute of Atomic and Molecular Sciences, Academia Sinica, Taipe 10617, Taiwan; 4 Advanced Optoelectronic Technology Center, National Cheng Kung University, Tainan 70101, Taiwan

**Keywords:** Substrate influence, Graphene, Raman lineshapes, Voigt fitting

## Abstract

We provide a new approach to identify the substrate influence on graphene surface. Distinguishing the substrate influences or the doping effects of charged impurities on graphene can be realized by optically probing the graphene surfaces, included the suspended and supported graphene. In this work, the line scan of Raman spectroscopy was performed across the graphene surface on the ordered square hole. Then, the bandwidths of G-band and 2D-band were fitted into the Voigt profile, a convolution of Gaussian and Lorentzian profiles. The bandwidths of Lorentzian parts were kept as constant whether it is the suspended and supported graphene. For the Gaussian part, the suspended graphene exhibits much greater Gaussian bandwidths than those of the supported graphene. It reveals that the doping effect on supported graphene is stronger than that of suspended graphene. Compared with the previous studies, we also used the peak positions of G bands, and *I*_2D_/*I*_G_ ratios to confirm that our method really works. For the suspended graphene, the peak positions of G band are downshifted with respect to supported graphene, and the *I*_2D_/*I*_G_ ratios of suspended graphene are larger than those of supported graphene. With data fitting into Voigt profile, one can find out the information behind the lineshapes.

## Background

Graphene has many unique and novel electrical and optical properties [1-3] because it is the thinnest sp^2^ allotrope of carbon arranged in a honeycomb lattice. Recent studies indicate that the remarkable carrier transport properties of suspended graphene with respect to supported graphene include temperature transport, magnetotransport, and conductivity [4-6]. The phonon modes of graphene and their effects on its properties due to the dopants and defects' effects are also different between suspended and supported graphene. These effects on its properties can be studied by Raman spectroscopy [7-9]. Raman spectroscopy has been extensively used to investigate the vibration properties of materials [10-13]. Recently, characterizing the band structure of graphene and the interactions of phonons has been applied as the powerful study method [14-18]. With the different effects influenced by doping and substrate, charged dopants produced by residual photoresist in the fabrication process are possibly induced by the deposition and also affect the substrate. According to relevant studies [19,20], the properties of metallic particles on graphene used as an electrode in graphene-based electronic devices can be understood clearly and suspended graphene is suitable to use to understand the effect of charged dopants on the substrate. In our previous works [21,22], we used polarized Raman spectroscopy to measure the strain effect on the suspended graphene. We fitted the spectra with triple-Lorentzian function and obtained three sub-2D peaks: 2D_+_, 2D_-_, and 2D_0._ In another work, we observed three sub-G peaks: G_+_, G_-_, and G_0_. The property of intensity of G_+_, G is similar as 2D_+_ and 2D peaks. The linewidth analysis with data fitting into pure Lorentzian and Voigt profiles had been applied two-photon transitions in atomic Cs [23,24], because of its elastic motion of atomic structures. The Voigt profile, a convolution of a Lorentzian and a Gaussian, is used to fit these Raman spectra of graphene.

In this work, the supported and suspended graphene were both fabricated by micromechanical cleavage, and then, they were identified as monolayer graphene by Raman spectroscopy and optical microscopy. The Raman signals of suspended and supported graphene can be measured and analyzed by probing the graphene surface which contains them. The peak positions of G band, the *I*_2D_/*I*_G_ ratio, and bandwidths of G band fitted with Voigt profile are obtained with the Raman measurements. Under our analysis, details about the effects of charged impurities on the substrate can be realized. About the strain effect or doping effect on graphene, some possible broadening mechanisms may still be responsible for deforming it, so we considered the Gaussian profile necessary.

## Methods

**Figure 1:**
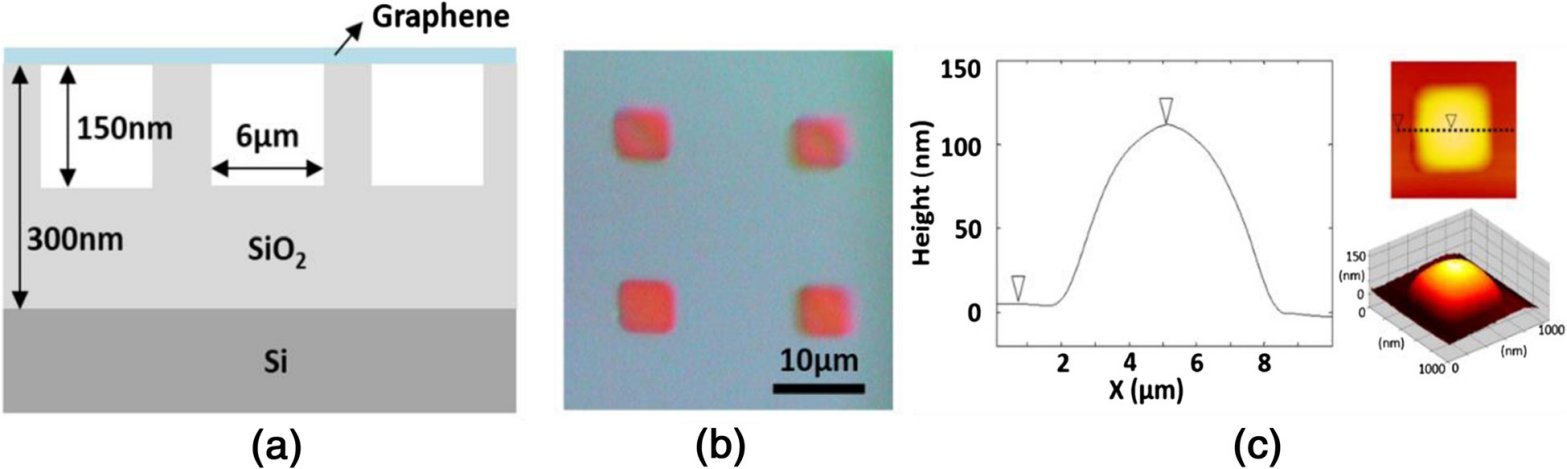
Structural illustration (a), optical image (b), and AFM image (c) and its cross section of suspended and supported graphene sample.

Suspended graphene was fabricated by mechanical exfoliation of graphene flakes onto an oxidized silicon wafer, and the illustration of that is shown in Figure 1a. First, ordered squares with areas of 6 μm^2^ were defined by photolithography on an oxidized silicon wafer with an oxide thickness of 300 nm. Reactive ion etching was then used to etch the squares to a depth of 150 nm. Micromechanical cleavage of highly ordered pyrolytic graphite was carried out using scotch tape to enable the suspended graphene flakes to be deposited over the indents. The thickness of the monolayer grapheme is about 0.35 nm. The optical image of suspended graphene, atomic forced microscopy (AFM) image, and its cross section are shown in Figure 1b,c. The surface of suspended graphene is like a hat, and the top of graphene surface can reach 100 nm high with respect to supported graphene. To identify the number of graphene layers and their properties, a micro-Raman microscope (Jobin Yvon iHR550, HORIBA Ltd., Kyoto, Japan) was utilized to obtain the Raman signals of monolayer graphene. A 632-nm He-Ne laser was the excitation light source. The polarization and power of the incident light were adjusted by a half-wave plate and a polarizer. The laser power was monitored by a power meter and kept constant as the measurements were made. The experimental conditions for Raman measurement were as follows. In order to avoid the local heating effect, the excited laser power on the graphene surface was 0.45 mW and the integration time was 180 s. The laser beam was focused by a × 50 objective lens (NA = 0.75) on the sample with a focal spot size of about 0.5 μm, representing the spatial resolution of the Raman system. Finally, the Raman scattering radiation was sent to a 55-cm spectrometer for spectral recording.

**Figure 2:**
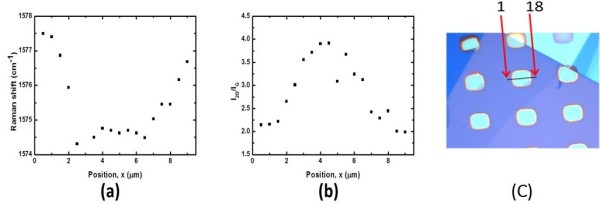
**Peak positions of G band and *I***_**2D**_**/*I***_**G **_**ratios by integrating their respect band. (a)** Raman positions of G band and **(b)***I*_2D_/*I*_G_ ratios of the probed area by scanning the mapping points on suspended graphene **(c)** shows the line mapping parameter.

To understand the unique properties of graphene surface covering on the different substrates, the Raman signals of G and 2D bands of graphene were obtained in these measurements. According to previous study [25], the *I*_2D_/*I*_G_ ratios and peak positions of G and 2D bands were various as graphene surface was doped by depositing silver nanoparticles on its surface. The *I*_2D_/*I*_G_ ratios and peak positions can be related to the doping, and the *I*_2D_/*I*_G_ ratio is more sensitive to the doping than is the peak shift. A lower *I*_2D_/*I*_G_ ratio is associated with a larger amount of charged impurities in graphene. Therefore, peak positions of G band and *I*_2D_/*I*_G_ ratios by integrating their respect band, G and 2D band, are obtained in Figure 2a,b. The horizontal axis is expressed as the positions of the focused laser which scanned across the graphene surface in the Raman measurement. The interval of line mapping points is set as 0.5 μm. Obvious frequency shift can be found at the G band peak positions between the suspended and supported graphene. The peak positions of G band of suspended and supported graphene are around 1,575 and 1,577 cm^-1^, and the *I*_2D_/*I*_G_ ratios of suspended and supported graphene are around 3.9 and 2.1. The upshift of the G band reflects doping with charged impurities. The peak position of the G band of the suspended graphene is redshifted comparing to that of supported graphene, consistent with the above expectations.

**Figure 3:**
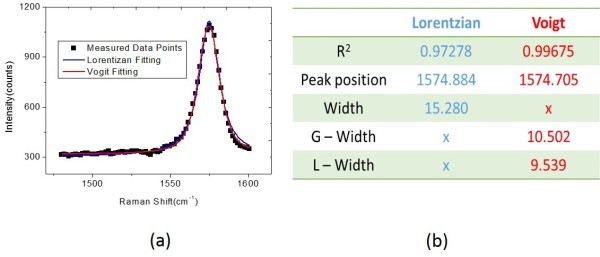
**The Raman spectrum of graphene and the related fitting parameter of the Raman spectra. (a)** The Raman spectrum (black line) of graphene, the Lorentzian-fitted profile (blue line), and the Voigt-fitted profile (red line). **(b)** The related fitting parameter of the Raman spectra.

The examination on G-band peak positions and the *I*_2D_/*I*_G_ ratios for monolayer graphene flake covering on different substrates can provide information of substrate effect. In the previous reviews, the bandwidths of G and 2D bands were usually fitted by Lorentzian function [26-29], because it just related to the lifetime broadening between the levels. However, the bandwidth broadening of G bands was clearly observed and deserved worth to be investigated. Here, we introduced that the Voigt profile, a convolution of a Lorentzian and a Gaussian, is suitable for fitting the transition linewidth and expressed [30-32] as

(1) $$\begin{array}{ll} V\left(\omega ,\Gamma ,\gamma \right) & =L\left(\omega ,\Gamma \right)*G\left(\omega ,\gamma \right)\\ & =\frac{\Gamma }{2\pi \gamma \sqrt{2\pi }}\overset{\infty }{\underset{-\infty }{\int }}\frac{\text{exp}\left(-\frac{x^{2}}{2\gamma^{2}}\right)}{{\left(\omega -x\right)}^{2}+\frac{\Gamma^{2}}{4}}\;\mathrm{dx}, \end{array}$$

where the Gaussian profile and Lorentzian profile are expressed as *G*(ω, γ) and *L*(ω, Γ), and γ and Γ are their bandwidths. In Figure 3a, the typical Raman spectrum (black line) of graphene was shown with the Lorentzian-fitted profile (blue line) and the Voigt-fitted profile (red line). The related fitting parameter of the Raman spectrum was showed in Figure 3b.

**Figure 4:**
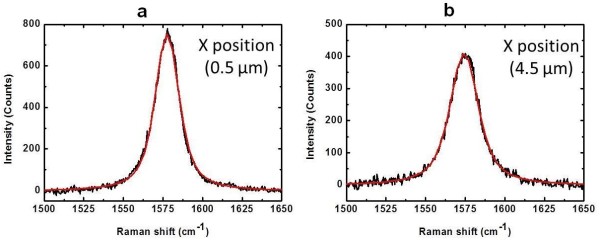
Raman spectra (black line) of (a) supported and (b) suspended graphene fitted by Voigt function (red line).

The bandwidth of Raman band was usually fitted and understood the situation of background of material by Gaussian function. Therefore, the G bands of supported and suspended graphene were fitted by Voigt profiles that give the Gaussian and Lorentzian profiles. The fitting results of Raman spectra of supported (*x* = 0.5 μm) and suspended (*x* = 4.5 μm) graphene by Voigt profile are shown in Figure 4a,b.

## Results and discussion

Based on the data fitting results, the analysis of measured point across the graphene surface, the bandwidths of Gaussian profiles and Lorentzian profiles given by Voigt fitting is presented in Figure 4a,b. The horizontal axis is expressed as the mapping points of the area which contains supported (edge area) and suspended graphene (center area).

**Figure 5:**
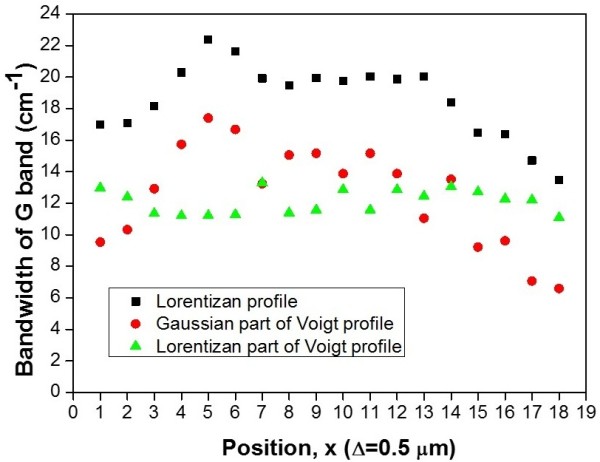
**Bandwidths of G band of the probed area by scanning the mapping points on suspended graphene.** By fitting with Voigt function contained (green triangle) Lorentzian part and (red circle) Gaussian part.

The Lorentzian bandwidths on the suspended and supported graphene are kept as 12.09 ± 0.76 cm^-1^. The Lorentzian bandwidth is mainly contributed by the natural linewidth and partly from the uncertainty of data fitting (0.3 cm^-1^) and instrumental uncertainty (0.9 cm^-1^). The natural linewidth is just linked with the phonon lifetimes between interaction levels. On the other hand, the Gaussian bandwidths of the suspended graphene exhibit a much higher than those of the supported graphene. Some mechanisms resulted in the Gaussian bandwidth broadening and the curve is consistent with the deformation of graphene surface. Other broadening mechanisms are related to the substrate effect and the local heating effect (Figure 5).

## Conclusions

Spectroscopic investigation on graphene of the interaction between phonons and electrons with the dopant or the substrate reveals a rich source of interesting physics. Raman signals of supported and suspended monolayer graphene were obtained. The peak positions of G bands, and *I*_2D_/*I*_G_ ratios, and bandwidths of G bands fitted with Voigt profiles were obtained under our analysis, and their different performances of suspended and supported graphene can be used to demonstrate the substrate influences and doping effects on graphene. The Gaussian bandwidths of those separated from Voigt profiles provide a new method to study the influence of the substrate and doping effect on graphene.

## Competing interests

National Science Council, Taiwan under contact no. NSC 101-2112-M-006-006 and NSC 102-2622-E-006-030-CC3.

## Authors' contributions

CHH, HL, and CWH carried on the experimental parts: the acquisition of data, and analysis and interpretation of data. YL took the analysis and interpretation of data, and also had been involved in revising the manuscript. FS and WW (Institute of Atomic and Molecular Sciences, Academia Sinica) prepared the samples, suspended graphene using by micromechanical method, and captured the OM and AFM images. HC, the corresponding author, had made substantial contributions to conception and design, and had been involved in drafting the manuscript and revising it critically for important intellectual content. All authors read and approved the final manuscript.
